# Intraoperative [^18^F]FDG flexible autoradiography for tumour margin assessment in breast-conserving surgery: a first-in-human multicentre feasibility study

**DOI:** 10.1186/s13550-021-00759-w

**Published:** 2021-03-18

**Authors:** Patriek A. G. T. Jurrius, Maarten R. Grootendorst, Marika Krotewicz, Massimiliano Cariati, Ashutosh Kothari, Neill Patani, Paulina Karcz, Monika Nagadowska, Kunal N. Vyas, Arnie Purushotham, Maria Turska-d’Amico

**Affiliations:** 1 School of Cancer and Pharmaceutical Sciences, King’s College London, London, United Kingdom; 2 Lightpoint Medical Ltd., London, United Kingdom; 3 Breast Cancer and Reconstructive Surgery Clinic, Maria Skłodowska-Curie Institute of Oncology, Warsaw, Poland; 4 Department of Breast Surgery, University College London Hospital, London, United Kingdom; 5 Department of Breast Surgery, Guy’s and St Thomas’ NHS Foundation Trust, London, United Kingdom; 6 Clinical Department of Endocrinology, Maria Skłodowska-Curie Institute of Oncology, Kraków, Poland; 7 Oncological and Reconstructive Surgery Clinic, Maria Skłodowska-Curie Memorial Cancer Centre and Institute of Oncology, Gliwice, Poland

**Keywords:** Breast cancer, Breast-conserving surgery, Re-operation rate, Margin assessment, Flexible autoradiography

## Abstract

**Introduction:**

In women undergoing breast-conserving surgery (BCS), 20–25% require a re-operation as a result of incomplete tumour resection. An intra-operative technique to assess tumour margins accurately would be a major advantage. A novel method for intraoperative margin assessment was developed by applying a thin flexible scintillating film to specimens—flexible autoradiography (FAR) imaging. A single-arm, multi-centre study was conducted to evaluate the feasibility of intraoperative [^18^F]FDG FAR for the assessment of tumour margins in BCS.

**Methods:**

Eighty-eight patients with invasive breast cancer undergoing BCS received ≤ 300 MBq of [^18^F]FDG 60–180 min pre-operatively. Following surgical excision, intraoperative FAR imaging was performed using the LightPath^®^ Imaging System. The first 16 patients were familiarisation patients; the remaining 72 patients were entered into the main study. FAR images were analysed post-operatively by three independent readers. Areas of increased signal intensity were marked, mean normalised radiances and tumour-to-tissue background (TBR) determined, agreement between histopathological margin status and FAR assessed and radiation dose to operating theatre staff measured. Subgroup analyses were performed for various covariates, with thresholds set based on ROC curves.

**Results:**

Data analysis was performed on 66 patients. Intraoperative margin assessment using FAR was completed on 385 margins with 46.2% sensitivity, 81.7% specificity, 8.1% PPV, 97.7% NPV and an overall accuracy of 80.5%, detecting both invasive carcinoma and DCIS. A subgroup analysis based on [^18^F]FDG activity present at time of imaging revealed an increased sensitivity (71.4%), PPV (9.3%) and NPV (98.4%) in the high-activity cohort with mean tumour radiance and TBR of 126.7 ± 45.7 photons/s/cm^2^/sr/MBq and 2.1 ± 0.5, respectively. Staff radiation exposure was low (38.2 ± 38.1 µSv).

**Conclusion:**

[^18^F]FDG FAR is a feasible and safe technique for intraoperative tumour margin assessment. Further improvements in diagnostic performance require optimising the method for scintillator positioning and/or the use of targeted radiopharmaceuticals.

*Trial registration*: Identifier: NCT02666079. Date of registration: 28 January 2016. URL: https://clinicaltrials.gov/ct2/show/NCT02666079.

*ISRCTN registry:* Reference: ISRCTN17778965. Date of registration: 11 February 2016. URL: http://www.isrctn.com/ISRCTN17778965.

## Introduction

The aim of breast conserving surgery (BCS) is to remove the cancer with an adequate margin of normal tissue (wide local excision—WLE), to ensure complete removal of the tumour with a good cosmetic outcome. Positive surgical margins represent a high risk for local recurrence following WLE and require further surgery to achieve clear margins. Previous studies have reported positive resection margins in 20–25% of the patients who underwent WLE [1–3]. Re-operation rates due to positive surgical margins following BCS reported in the literature range from 17 to 68% [2]. In addition to repeat operations, the consequences of positive surgical margins potentially include delayed adjuvant treatment, poorer cosmesis, emotional distress and financial cost [1, 2].

The technologies that are clinically available for assessment of surgical margins intraoperatively have limitations, and no single technique has been accepted as standard of care due to issues of practicality, increased surgery time, costs and limited sensitivity and specificity [4, 5]. To address the limitations of existing techniques, there are a variety of techniques under development [4, 6, 7].

**Fig. 1:**
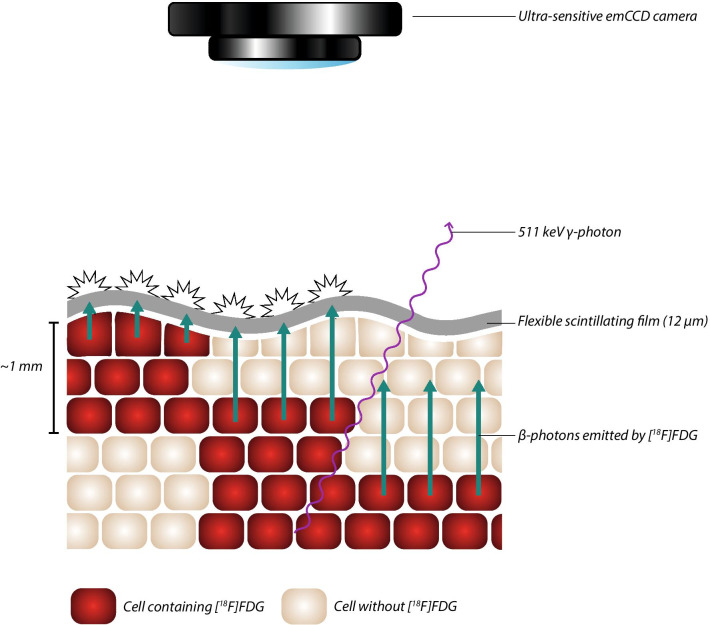
Schematic representation of FAR imaging using a flexible scintillating film. Tumour cells containing a PET-radiotracer (e.g. [^18^F]FDG) emit β^+^-particles which are converted to scintillations by the flexible scintillator. As β^+^-particles travel a limited distance in tissue, [^18^F]FDG containing cells are detected up to approximately 1 mm in tissue. The scintillations are measured by an ultra-sensitive emCCD camera. The flexible scintillators 12 µm thickness makes the scintillator insensitive to 511 keV ɣ-photons

This study introduces a novel approach on flexible radioluminescence (Flex-RLI), a technique introduced by Jenkins et al*.* and proposed for FDG-guided surgery by King et al*.* [8, 9]. Similarly, flexible autoradiography (FAR) relies on the detection of scintillations. However, in FAR these scintillations are produced by a micrometres-thick flexible scintillating film draped over an excised specimen (Fig. 1) rather than a millimetre-thick gel scintillator. The main merit of a flexible scintillating film compared with traditional rigid autoradiography techniques is its property to conform to the shape of the excised specimen, thereby maximising the contact area, resulting in an enhanced signal intensity, improved signal-to-noise ratio (SNR) and minimal signal attenuation due to distance (variations) between tumour and scintillator [8–10]. This should reduce image acquisition times and improve diagnostic accuracy. [^18^F]FDG FAR provides a signal from cells containing [^18^F]FDG at a depth of up to _~_1 mm, as β^+^-particles travel approximately 1 mm through human tissue [11] (Fig. 1). This correlates well with current breast cancer margin definitions [10, 12].

The aim of this first-in-human study was to assess the feasibility of [^18^F]FDG FAR for intraoperative tumour margin assessment in patients undergoing WLE for invasive breast cancer.

## Materials and methods

### Trial set-up and patient population

This was a prospective, single-arm, multi-centre feasibility study. All required regulatory approvals were obtained prior to patient recruitment. Between 2 February 2018 and 2 July 2019, women ≥ 18 years of age diagnosed with biopsy confirmed invasive breast cancer and scheduled to undergo primary WLE with or without sentinel lymph node biopsy (SLNB) or axillary lymph node dissection (ALND) were recruited at three study sites in Poland. Exclusion criteria were ductal carcinoma in situ (DCIS), pleomorphic lobular carcinoma in situ (LCIS), surgery or radiotherapy to the ipsilateral breast, impalpable lesions scheduled to have radio-guided occult lesion localisation (ROLL), neoadjuvant chemotherapy, known hypersensitivity to [^18^F]FDG, pregnancy, lactation, an active history of other cancer or other health issues compromising study participation. Women of childbearing age were required to have a negative beta-HCG qualitative test result, a history of surgical sterilisation or amenorrhea in the past 12 months.

### Radiopharmaceutical administration

Patients scheduled for SLNB received up to 150 MBq technetium-99m-labelled nanocolloid (^99m^Tc-nanocolloid) administered according to local practice on the morning of surgery. This activity level was based on a previous study performed by Grootendorst et al. [6]. A potentially higher injected ^99m^Tc-nanocolloid activity aimed to reduce the effect of [^18^F]FDG crosstalk due to down-scatter of 511 keV fluorine-18 ɣ-photons into the technetium-99m ɣ-probe energy window. Following the ^99m^Tc-nanocolloid injection, all trial patients received an intravenous injection of [^18^F]FDG ≤ 5 MBq/kg, with a maximum of 300 MBq, 60–180 min prior to intraoperative FAR imaging. The administered activity was determined on a per site and per patient basis.

### Surgery

Following induction of anaesthesia, patients due to undergo SLNB received an intraoperative blue dye injection as per local guidelines and SLNs were identified by using a hand-held ɣ-probe and blue discoloration of lymph nodes. In the majority of cases, WLE was performed prior to the SLNB/ALND, to ensure a minimum signal intensity loss from radioactive decay of [^18^F]FDG in the time window between injection and FAR imaging. The WLE specimen was removed, and sutures/surgical clips were placed on the specimen to record the anatomical orientation.

### FAR imaging system

The LightPath^®^ Imaging System (Lightpoint Medical Ltd., Chesham, UK), an in vitro diagnostic device, was used to visualise the location and distribution of [^18^F]FDG using FAR. This system is further described by Ciarrocchi et al*.* [13]. A 12-µm-thick flexible scintillating film was used as an accessory to the LightPath^®^ System (Fig. 1) and consisted of a multilayer sandwich construction as follows: 3 µm of mylar, 6 µm of P43 scintillating phosphor and 3 µm of mylar. The thinness of these layers made the scintillator insensitive to the ^18^F-FDG 511 keV ɣ-photons [10].

Images were acquired using a 300 s acquisition time and 8 × 8 pixel binning (pixel resolution 938 μm). These imaging settings were based on the results from the clinical [^18^F]FDG Cerenkov luminescence imaging (CLI) study in breast cancer [6], and an in vitro FAR study performed with the LightPath^®^ Imaging System [14].

To reject the discrete signals from high-energy annihilation ɣ-photons, three emCCD frames were taken with an acquisition time of 100 s each. The emCCD frames were then combined into a single image by applying a spatial–temporal median filter (3 × 3 pixels × 3 time points). A Gaussian smoothing filter (*σ* = 3 pixels) was applied, and the resultant image was scaled and translated for overlay display on top of the reference image. The resulting merged image was called the FAR image.

### Intraoperative imaging

**Fig. 2:**
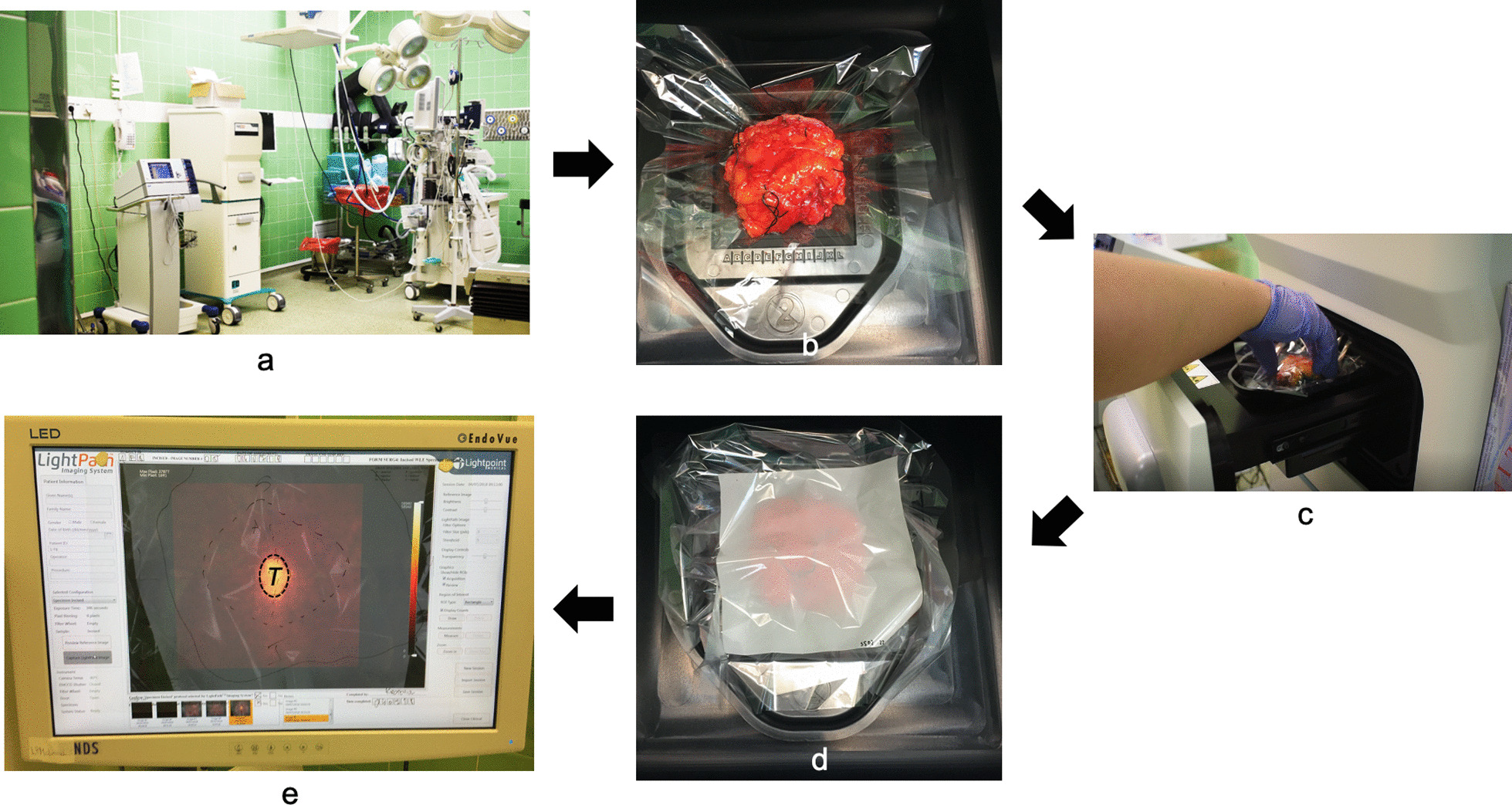
Workflow of intraoperative FAR imaging. **a** The LightPath^®^ Imaging System was located within the operating theatre. **b** Immediately following dissection, the intact WLE specimen was placed in a disposable specimen tray. **c** The specimen tray and specimen were loaded into the light-tight specimen chamber, and a photographic reference image was acquired to confirm that the specimen was correctly positioned. The specimen contours were drawn on a transparent CRF, and each tumour margin was annotated (not shown). **d** A 5 μm Mylar separator sheet and a flexible scintillator film were draped over the specimen, and a FAR image was acquired. (e) FAR image of WLE specimen shows elevated tumour radiance. The transparent CRF attached to the LightPath^®^ monitor shows the specimen contours, tumour margin borders, the incision line and the location of the primary tumour (T) in the area of the elevated radiance

Following surgical excision and orientation, the WLE specimen was positioned in a disposable specimen tray, inserted in the imaging chamber and a reference image was acquired. A transparent plastic sheet was attached to the LightPath^®^ monitor, and based on the position of the specimen on the reference image, the specimen contours and tumour margins were outlined with a marker pen (Fig. 2). The specimen was then draped with a 5 μm Mylar separator sheet and a flexible scintillator film, and a FAR image acquired. The separator sheet acted as a protective layer to prevent contamination of the scintillator film from biological tissue and fluids. The aforementioned steps were repeated with the WLE specimen reorientated, placing the previously occult margins in the field of view. All images were acquired within 60–180 min following [^18^F]FDG administration. Upon completion of FAR imaging, the surgical specimens were sent for routine histopathology assessment.

To minimise the impact of any operator bias, training on the use of the LightPath^®^ system and FAR imaging was provided including positioning of the Mylar separator sheet and flexible scintillator film, prior to the start of the clinical trial at each study site. In addition, a representative of Lightpoint Medical Ltd. attended the familiarisation patient for each surgeon for hands-on training during clinical use. The first study patient of each surgeon was considered a familiarisation patient for the surgeon to become acquainted with FAR imaging and the intraoperative study procedures. These familiarisation patients were excluded from the analysis dataset.

### Radiation exposure

Prior to commencement of the study, staff at all study sites received radiation safety training as per local policies and procedures. At the start of each surgical procedure, designated staff members were issued with electronic personal radiation dose (EPD) monitors, worn in the anterior top pocket of the clothing. Ring dosimeters for the extremities were worn depending on local requirements. Thorough contamination monitoring of equipment, rooms, waste and staff was completed after each use, to ensure that potential radioactive contamination was identified and appropriately managed.

### Histopathology

Histopathological analysis was performed according to local practice with reporting on tumour characteristics, invasive and in situ components and distance from tumour to all 6 margins (anterior, posterior, lateral, medial, superior and inferior). Local margin definitions were used as follows: a positive margin for invasive cancer was defined as tumour cells at the margin (= 0 mm) at site 1 and 2, and tumour cells < 1 mm of margin at site 3. For DCIS, the definition was 0 mm (site 1 and 2) and < 2 mm (site 3). The histopathologists were blinded to the intraoperative FAR findings.

### Image analysis

Following acquisition, the FAR image was assessed by the surgeon during surgery and post-operatively by three independent breast surgeons, the central readers (Central Read). It was left to the operating surgeon’s discretion to use the FAR images for clinical decision-making. The Central Read was performed post-operatively to provide a controlled and standardised analysis environment; central readers were blinded to the intraoperative FAR and post-operative histopathology results. The central readers visually analysed the FAR image in the LightPath^®^ software (version 2.0.20) to identify areas of increased signal intensity called “hotspots”. To distinguish tumour hotspots from artefacts, tumour hotspots were defined as a focal area of more than 1 mm in diameter that displayed an increased signal intensity over tissue background. Artefacts were clearly identifiable by their morphology (Additional file 1: Fig. 1). In this manner, hotspots were classified as either tumour hotspots or artefacts. All readers were taught to recognise image artefacts as part of the formal training that took place prior to image analysis. A tumour hotspot on the intact WLE images resulted in a tumour margin being classified as positive on FAR. Each central reader analysed approximately one-third of the total number of images.

The presence of a tumour hotspot indicated the tumour was close to the surface as the positrons from [^18^F]FDG travel only approximately 1 mm in tissue [11]. Upon completion of image analysis, the margin status for each specimen margin on FAR was compared to the margin status as determined by local standard-of-care histopathology examination at each site. FAR margin definition histopathology margin definition may result in false positives and false negatives.

**Fig. 3:**
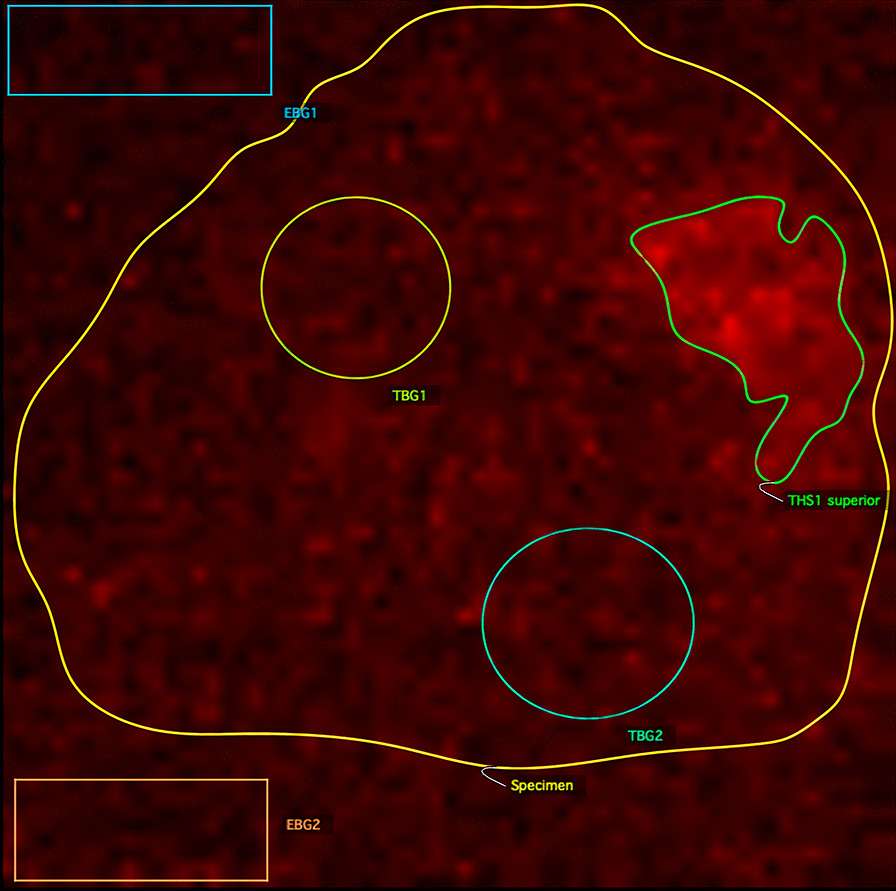
A WLE excision specimen (outlined in yellow) analysed in OsiriX Lite version 11. ROIs are drawn to quantify the signal intensity of the empty background (EBG), tissue background (TBG) and tumour hotspot (THS). The area of increased signal intensity at the superior margin (THS1 superior) contained invasive carcinoma on histopathology analysis

The emCCD images were quantitatively analysed by an independent researcher/surgical fellow blinded to the histopathology results using OsiriX Lite version 11 (Pixmeo SARL, Geneva, Switzerland). Based on the findings from the Central Read, regions of interest (ROIs) were manually drawn to mark areas of increased signal intensity and background signal within and outside the specimen’s contours (Fig. 3). The location, mean, minimum and maximum radiance (photons/s/cm^2^/sr/MBq) were recorded for each ROI. Tumour-to-tissue background ratios (TBR) were calculated. The radiance was normalised by the decay-corrected [^18^F]FDG activity at time of FAR imaging to make the ROIs comparable between images taken at different time points.

Images acquired outside the 60–180 min time window or containing an image acquisition artefact were excluded from analysis.

### Statistical analysis

The diagnostic accuracy of FAR was assessed by calculating the sensitivity, specificity, positive predicting value (PPV), negative predictive value (NPV) and overall accuracy. Subgroup analysis was performed to assess the effect of study site, tumour characteristics, time of imaging and injected [^18^F]FDG activity decay corrected for the time of FAR image acquisition on diagnostic accuracy. Thresholds for decay corrected injected [^18^F]FDG activity and time between [^18^F]FDG injection and intraoperative FAR imaging were determined by ROC analysis.

## Results

### Patients and pharmaceutical characteristics

Intraoperative FAR images were acquired from 88 patients; 16 patients as part of the familiarisation population and the subsequent 72 patients entered into the main study. A total of 6 patients in the main study were excluded after surgery: 2 patients had FAR imaging outside the 60–180 min time window, one patient only had in situ breast cancer on post-operative histopathology, and 3 patients had FAR images that were unevaluable due to the presence of an image processing artefact. Hence, a total of 66 patients were included in the analysis dataset. Patient demographics and tumour characteristics are shown in Table 1.

**Table 1:** Patient demographics and tumour characteristics

Variable	Category	Results	Missing data (*n* (%))
Age [years]	*n*	66 (100%)	0 (0.0%)
	Mean (SD)	60.0 (9.4)	
	Median [Q1–Q3]	61.0 [56.0–66.0]	
	Min–max	28.0–78.0	
BMI [kg/m^2^]	*n*	66 (100%)	0 (0.0%)
	Mean (SD)	27.4 (4.7)	
	Median [Q1–Q3]	27.0 [23.5–30.1]	
	Min–max	19.1–38.5	
Histological tumour grade		*n* = 62 (93.9%)	4 (6.1%)
	G1	22 (35.5%)	
	G2	24 (38.7%)	
	G3	16 (25.8%)	
T-classification		*n* = 64 (97.0%)	2 (3.0%)
	Tis	0 (0.0)	
	T1	49 (76.6%)	
	T2	15 (23.4%)	
N-classification		*n* = 63 (95.5%)	3 (4.5%)
	N0	54 (85.7%)	
	N1	8 (12.7%)	
	N2	1 (1.6%)	
M-classification	M0	*n* = 61 (92.4%)	5 (7.6%)
Tumour size (invasive [mm])	*n*	64 (97.0%)	2 (3.0%)
	mean (SD)	16.0 (7.2)	
	Median [Q1Q3]	14.0 [12.0–21.0]	
	Min–max	2.00–35.00	
Tumour type		*n* = 52 (78.8%)	14 (21.2%)
	Ductal/no special type (NST)	38 (73.1%)	
	Lobular	3 (5.8%)	
	Mucinous	2 (3.8%)	
	Tubular/cribriform	1 (1.9%)	
	Medullary	1 (1.9%)	
	Micropapillary	2 (3.8%)	
	Mixed	5 (9.6%)	1 (1.9%)
	Apocrine differentiation	2 3.8%)	
	Neuroendocrine differentiation	2 (3.8%)	
Associated DCIS		*n* = 42 (63.6%)	
	Low grade	14 (33.3%)	
	Intermediate grade	22 (52.4%)	
	High grade	6 (14.3%)	
Lymphovascular invasion		*n* = 65 (98.5%)	1 (1.5%)
	Present	8 (12.3%)	
	Absent	57 (87.7%)	
Histological receptor status		*n* = 64 (97.0%)	2 (3.0%)
	ER positive	58 (90.6%)	
	PR positive	47 (73.4%)	
	HER2 positive	4 (6.3%)	

SD, standard deviation; ER, oestrogen receptor; PR, progesterone receptor; HER2, human epidermal growth factor receptor 2

The mean administered [^18^F]FDG activity per patient was 246.7 ± 48.6 MBq (range: 185.0–334.0 MBq). Intraoperative FAR images of intact WLE specimens were acquired 143.9 ± 20.5 post-injection (p.i.).

### FAR imaging

In total, 385 margins in 66 patients were assessed on both FAR and histopathology. A total of 13 positive margins were found on histopathology assessment in 9 patients (5 × invasive carcinoma, 4 × DCIS and 4 × invasive carcinoma + DCIS). FAR was able to accurately detect 6 of the 13 histopathological positive margins in 4 patients (true positive) − 2 × invasive carcinoma, 2 × DCIS and 2 × invasive carcinoma + DCIS. In all, 7 of 13 margins were missed in 5 patients (false negative) − 3 × invasive carcinoma, 2 × DCIS and 2 × invasive carcinoma + DCIS.

Of the 372 histopathological negative margins in 55 patients, FAR accurately detected 304 margins (true negative) and wrongly classified 68 margins as positive (false positive).

The average diagnostic accuracy of FAR for imaging intact WLE specimens was 46.2% sensitivity, 81.7% specificity, 8.1% PPV, 97.7% NPV and an overall accuracy of 80.5%.

### Subgroup analysis

**Fig. 4:**
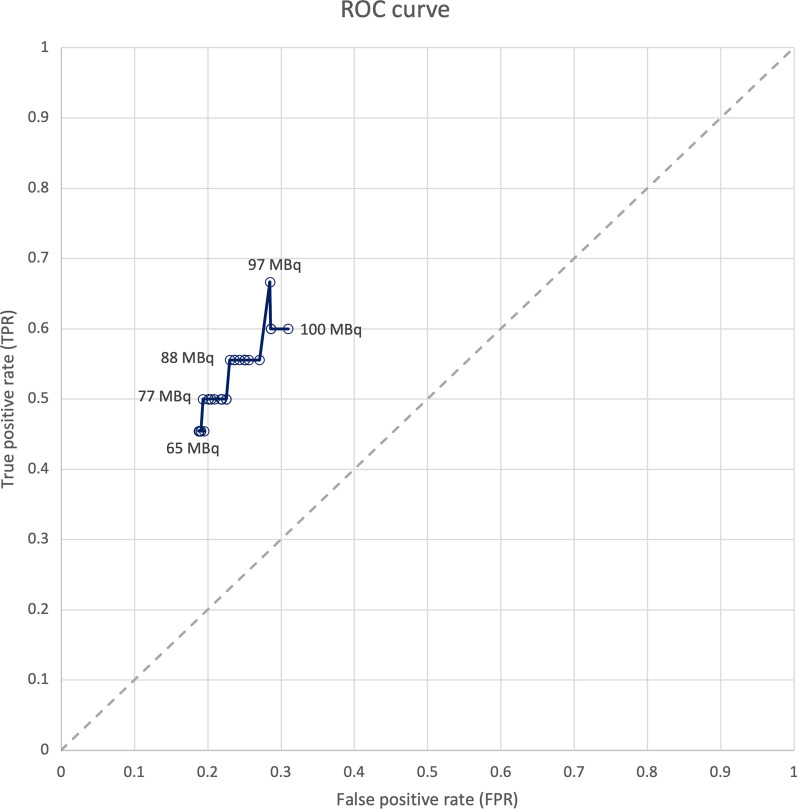
Receiver operator curve (ROC) of intraoperative FAR performance on intact WLE specimen for various decay corrected injected [^18^F]FDG activity thresholds with increments of 1 MBq

A subgroup analysis divided woman into a high (*n* = 31) and a low (*n* = 35) injected activity of [^18^F]FDG decay corrected for the time at which FAR images were acquired. The subdivision threshold for the decay corrected injected [^18^F]FDG activity was determined at 97.0 MBq, as per ROC analysis (Fig. 4).

Women in the high-activity cohort received on average 283.7 ± 31.4 MBq vs. 213.9 ± 35.9 MBq in the low-activity cohort. The difference of mean injected [^18^F]FDG doses between these two cohorts is statistically significant (p < 0.001). In both cohorts, FAR imaging of the intact WLE specimen was performed approximately 145 ± 21 min p.i.

Of the 6 true positive margins, 5 (83.3%) were identified in the high-activity cohort (Table 2). In this high-activity cohort, the areas of increased signal intensities at the tumour-containing margins showed a mean normalised radiance of 126.73 ± 45.71 photons/s/cm^2^/sr/MBq on FAR imaging, whereas the mean normalised radiance from tumour negative margins was 56.5 ± 25.3 photons/s/cm^2^/sr/MBq in those images. The TBR was 2.1 ± 0.5 (range 1.1–2.6). The normalised tumour radiance, background radiance and TBR in the low-activity cohort, based on the single image containing a true positive margin, were 145.2 photons/s/cm^2^/sr/MBq, 68.9 photons/s/cm^2^/sr/MBq and 2.1, respectively. Five out of 7 (71%) false-negative margins were found in the low-activity cohort. Of the 68 false-positive margins, 49 were found in the high-activity cohort (72%).

**Table 2:** Confusion matrix of FAR imaging for intraoperative margin assessment of intact WLE specimens compared to gold standard histopathology. Results are shown for the overall cohort as well as for subgroups based on the decay corrected [^18^F]FDG activity (threshold 97.0 MBq) and invasive tumour grade

Cohort		Histopathologically positive	Histopathologically negative
High	FAR positive	5	49
	FAR negative	2	125
Low	FAR positive	1	19
	FAR negative	5	179
Grade 1	FAR positive	3	18
	FAR negative	1	110
Grade 2	FAR positive	3	33
	FAR negative	6	99
Grade 2	FAR positive	0	15
	FAR negative		
		0	79
Overall	FAR positive	6	68
	FAR negative	7	304

An increased sensitivity, PPV and NPV (71.4%, 9.3% and 98.4%, respectively) were observed for the high-activity cohort (Table 3). Comparing the low and high activity cohorts showed that the true-positive rate increased by a factor of 5.6 for the high-activity cohort and the false-positive rate increased by a factor of 2.9.

**Table 3:** Diagnostic accuracy of FAR for intraoperative margin assessment of intact WLE specimens compared to histopathology for a high decay corrected [^18^F]FDG activity versus a low decay corrected [^18^F]FDG activity at time of FAR imaging as well as for the whole study population

	High-activity cohort (*n* = 181 margins)	Low-activity cohort (*n* = 204 margins)	Whole population (*n* = 385 margins)
Sensitivity	71.4% (29.04–96.33%)	16.7% (0.42–64.12%)	46.2% (19.22–74.87%)
Specificity	71.8% (64.53–78.38%)	90.4% (85.42–94.12%)	81.7 (77.41–85.52%)
PPV	9.3% (5.69–14.71%)	5.0% (0.83–24.88%)	8.1 (4.51–14.15%)
NPV	98.4% (95.07–99.51%)	97.3% (96.15–98.09%)	97.7 (96.32–98.63%)
Overall accuracy	71.8% (64.67–78.25%)	88.2% (83.00–92.31%)	80.5 (76.20–84.36%)

For the subgroup analysis on tumour grade, the overall study cohort consisted of 22, 24, and 16 patients with a grade 1, grade 2 and grade 3 tumour, respectively, and 4 patients with no tumour grade recorded. For grade 3, no histopathologically positive margins were found; hence, no diagnostic performance could be established for this subgroup. Comparing grade 1 vs grade 2 tumours and the overall study cohort, an improved diagnostic performance for grade 1 was observed (sensitivity 75.0%, specificity 85.9%, PPV 14.3%, NPV 99.1%, overall accuracy 85.6%) (Table 2).

Analyses for the other subgroups were inconclusive (Additional file 2: Table 1 and, Additional file 3: Table 2).

### Staff radiation exposure and adverse events

The mean individual whole-body effective radiation dose and finger dose as measured by theatre staff’s EPD and ring dosimeters were 38.2 ± 38.1 µSv and 59.9 ± 29.1 µSv, respectively (Table 4). Anaesthetic technicians received the lowest (20.0 ± 20.7 µSv) and the operating surgeons the highest mean whole-body dose (61.8 ± 45.5 µSv). The assisting surgeons, scrub nurses and anaesthesiologists received 54.8 ± 48.9 µSv, 33.4 ± 32.5 µSv and 26.4 ± 22.4 µSv, respectively (Table 4). These measured values for whole-body and finger radiation dose are in agreement with exposures described in literature and well below the regulatory thresholds as set out in Regulation 20 of the Ionizing Radiations Regulations 1999, the US Nuclear Regulatory Commission 1991 and the International Commission on Radiological Protection 1992 [15–19].

**Table 4:** Radiation dose from [^18^F]FDG and ^99m^Tc-nanocolloid as received by operating theatre per procedure for each staff groups

Variable	Category	Total	Operating surgeon	Assisting surgeon	Scrub nurse	Anaesthesiologist	Anaesthetic technician	Missing data
Number of staff members	*n*	393	66	63	132	66	66	
Measured EPD dose [µSv]	*n*	388	66	62	128	66	66	5 (1.3%)
	Mean (SD)	38.2 (38.1)	61.8 (45.5)	54.8 (48.9)	33.4 (32.5)	26.4 (22.4)	20.0 (20.7)	
	Median [Q1–Q3]	27.0 [11.0–56.0]	56.5 [38.0–74.0]	44.5 [27.5–66.0]	16.0 [4.0–59.0]	22.0 [16.0–28.8]	14.0 [10.0–20.0]	
	Min–max	0.0–335.0	0.0–335.0	3.0–262.0	0.0–140.0	3.0–135.0	3.0–113.0	
Finger badge dose received "Left" [µSv]	*n*	104	32	29	23	20	0	289 (73.5%)
	Mean (SD)	57.6 (26.8)	72.9 (21.2)	53.8 (17.9)	64.6 (33.0)	31.2 (15.4)		
	Median [Q1–Q3]	58.0 [38.0–75.0]	75.0 [58.0–94.3]	48.3 [38.0–66.0]	64.0 [30.8–91.4]	20.0 [16.7–45.0]		
	Min–max	16.7–104.0	43.3–104.0	33.3–82.9	16.7–100.0	16.7–64.0		
Finger badge dose received "Right" [µSv]	*n*	99	33	33	27	6	0	295 (75.1%)
	Mean (SD)	62.2 (31.3)	61.9 (30.4)	63.9 (25.8)	51.5 (13.0)	101.7 (75.1)		
	Median [Q1–Q3]	60.0 [53.0–70.0]	60.0 [56.0–60.0]	70.0 [53.0–70.0]	60.0 [46.5–60.0]	99.2 [33.3–165.0]		
	Min–max	29.0–220.0	33.3–220.0	29.0–145.0	29.0–60.0	33.3–180.0		

There were no device-related or radioactive contamination-related adverse events.

## Discussion

This first-in-human multicentre single-arm study evaluated the feasibility and performance of [^18^F]FDG FAR for the intraoperative assessment of excision margins in patients with invasive breast cancer undergoing BCS. Intraoperative FAR could be performed and assessed in 66 patients, with 66 intact WLE specimens included in the analysis. When compared to gold standard histopathology, the diagnostic accuracy of FAR provided sensitivity 46.2%, specificity 81.7%, PPV 8.1%, NPV 97.7% and overall accuracy 80.5% for margin assessment on intact WLE specimen images.

Subgroup analysis revealed improved sensitivity, PPV and NPV for women receiving a higher [^18^F]FDG activity. For intraoperative margin assessment in breast conserving surgery, high sensitivity is more important than a low false-positive rate to minimise the risk missing involved margins. An involved margin would require a reoperation whereas a false-positive margin results in the excision of a several millimetre-thick cavity shaving during the initial surgery, which has limited cosmetic impact. Based on this and the subgroup analysis, an [^18^F]FDG activity decay corrected for the time between injection and FAR image acquisition of at least 97 MBq is recommended. This corresponds to an [^18^F]FDG activity of 242 MBq administered 145 min prior to FAR imaging for the clinical workflow as demonstrated in this study.

The apparent improved performance of [^18^F]FDG FAR imaging for grade 1 is not understood and in contrast to the available PET literature on [^18^F]FDG uptake in relation to breast cancer grade [11–14]. This finding may be due to statistical limitations of the dataset.

Detection rates were similar for both invasive carcinomas (2 out of 5 margins) and DCIS (2 out of 4 margins) at the margin.

Exposure results demonstrated safety for all staff with a mean individual whole-body and finger effective radiation exposure of 38.2 ± 38.1 µSv and 59.9 ± 29.1 µSv, respectively.

Surgeons using [^18^F]FDG micro-PET/CT for intraoperative margins assessment have achieved similar sensitivity and specificity compared to the high-activity FAR cohort. However, for [^18^F]FDG micro-PET/CT injection-imaging time was longer (230 ± 57 min) as were the image acquisition times (33–43 min) [7].

The FAR signal intensity in the current clinical study is markedly lower than expected based on the in vitro LightPath^®^ FAR study by Olde Heuvel et al*.* [20]. Firstly, in a clinical setting the positrons are attenuated by biological tissue which reduces the number of scintillation photons and consequently the detectable radiance on FAR. In the in vitro experiment from Olde Heuvel et al., no tissue-mimicking material was used, thus overestimating the expected signal gain from in vivo FAR. Secondly, the operators of the FAR system indicated that it was challenging to accurately position the flexible scintillator film on the separator sheet and specimen. Because of their material properties, there was suboptimal adhesion between the scintillator film and the separator sheet, and as a result the scintillator film did not fully conform to the shape of the tissue specimen, leaving gaps between the scintillator film and the tissue’s surface. Inherently, this will have had a negative impact on the signal intensity and spatial resolution of the FAR image as the required travel distance for the β^+^-particles to reach the scintillator will have increased. Future work should focus on more accurately determining the minimal detectable activity level for successful use of FAR in a clinical setting and developing methods to enable more accurate and robust scintillator positioning.

### Limitations

The flexible scintillating film is semi-opaque, thus obscuring the white light specimen reference image, impeding accurate correlation of the FAR signal to the exact anatomical location on the specimen. Moreover, breast specimens “flatten” between excision and histopathology analysis, which changes the mean tissue volume and height by 30% and 46%, respectively [21]. Intraoperative marking of areas with increased signal intensity, e.g. a suture, would allow better correlation between FAR and histopathology. Furthermore, an increased ^99m^Tc-nanocolloid activity injected intra- or peri-tumourally, as done at 2 study sites, is likely to result in a higher ^99m^Tc-nanocolloid activity in the excised specimen. As ^99m^Tc-nanocolloid emits lower energy ɣ-particles than [^18^F]FDG (144 vs 511 keV), high concentrations of ^99m^Tc-nanocolloid may have resulted in optical signals from the flexible scintillator film. These factors could have contributed to inaccuracies in correlating the margin status on FAR with histopathology and the non-tumour related hotspots, thus partially explaining the substantial number of false-positive findings. Yet, the exact cause requires further investigation.

The recruitment period was limited by the duration of the grant funding, thereby limiting the patient recruitment figures. This has resulted in a limited number of true positive margins, impacting on the ability to evaluate the diagnostic accuracy of FAR.

[^18^F]FDG is considered a suboptimal radiopharmaceutical for imaging breast cancer as it is non-specific with substantial variations in uptake between breast tumour characteristics and breast cancer subgroups [22–26]. The use of tumour-specific tracers, targeting, for example, ER (e.g. [^18^F]FES), HER2 (e.g. [^68^Ga]ABY-025), PARP (e.g. [^18^F]SuPAR), FAP (e.g. [^68^Ga]FAPI) or GRPR (e.g. [^68^Ga]RM2) [27–31] are likely to further improve the diagnostic accuracy of FAR. An advantage of using Galium-68 labelled tracers for FAR is a 2 × stronger signal intensity compared to Fluorine-18 [20]. A clinical trial evaluating [^68^Ga]RM2 FAR in ER + patients is imminent (EudraCT number: 2017–003,212-39).

## Conclusion

[^18^F]FDG FAR has proven to be a safe and feasible technique for intraoperative assessment of excised surgical specimens in breast-conserving surgery. Positive margins containing both invasive cancer as well as DCIS were detected successfully. However, the sensitivity and PPV remained suboptimal due to the low number of true-positive margins and a substantial number of false positives. An improved sensitivity was seen in patients who received a higher [^18^F]FDG activity. Further improvements in diagnostic accuracy require optimising the method for scintillator positioning and/or the use of more tumour specific radiopharmaceuticals.

## Supplementary Information

**Additional file 1: Fig 1**. Example of LightPath^®^ images. (**a**) Intact WLE image from familiarisation population showing two tumour hotspots (white arrows). (**b**) Incised WLE image with a gamma strike artefact (white arrow). A gamma strike is an extremely bright pixel, typically of well-defined circular shape, and with a streak of horizontal pixels resembling a “comet tail”. Note that in this image the transparency slider was positioned halfway so that both the emCCD image and photographic reference image can be seen. (**c**) Intact WLE image showing a white square artefact. The image shows a very high, uniform brightness which prevents the identification of any details within the image. (**d**) Lymph node image showing a ring artefact. The dark can be seen. (**c**) Intact WLE image showing a white square artefact. The image shows a very high, uniform brightness which prevents the identification of any details within the image. (**d**) Lymph node image showing a ring artefact. The dark rings cover any relevant structure within that area, thus making the image unevaluable.

**Additional file 2: Table 1**. Confusion matrix of FAR imaging for intraoperative margin assessment of intact WLE specimens compared to gold standard histopathology. The patients were divided into two subgroups based on the time between [^18^F]FDG injection and intraoperative FAR imaging (threshold: 158 min). The mean injection to imaging times between the two subgroups was not statistically significant (p < 0.353).

**Additional file 3: Table 2**. Confusion matrix of FAR imaging for intraoperative margin assessment of intact WLE specimens compared to gold standard histopathology. The patients were divided into three subgroups based on T-classification.

## Data Availability

The datasets generated and analysed during the current study are available from the corresponding author on reasonable request.
