# Supplementary figures and images for: Intraoperative [18F]FDG flexible autoradiography for tumour margin assessment in breast-conserving surgery: a first-in-human multicentre feasibility study
Source: EJNMMI Res. 2021 Mar 18;11:28. doi: 10.1186/s13550-021-00759-w (PMC7973336; doi:10.1186/s13550-021-00759-w)

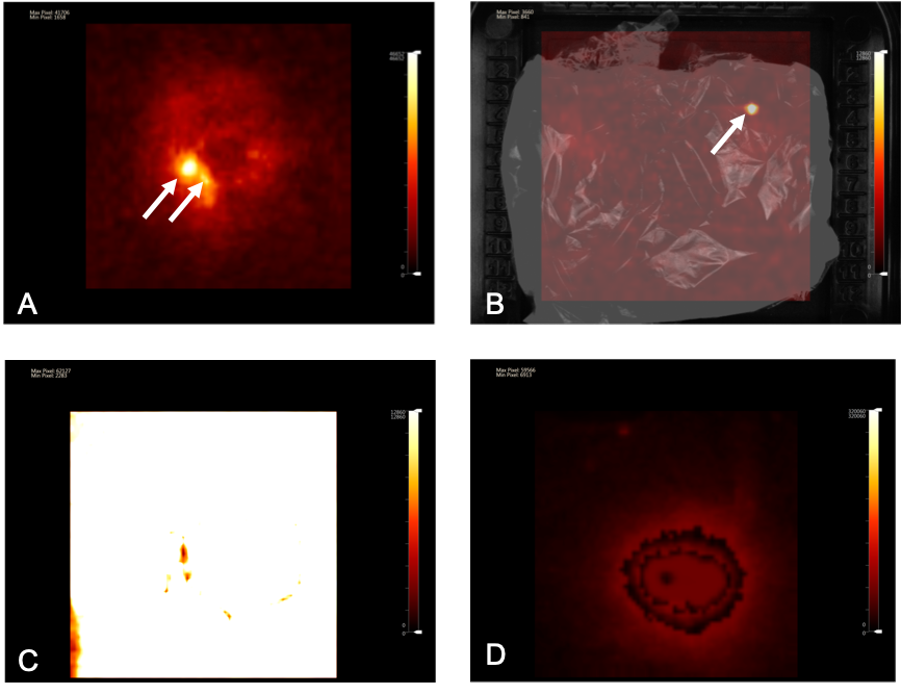

Supplement: Supplementary file 1 — Additional file 1: Fig 1. Example of LightPath® images. (a) Intact WLE image from familiarisation population showing two tumour hotspots (white arrows). (b) Incised WLE image with a gamma strike artefact (white arrow). A gamma strike is an extremely bright pixel, typically of well-defined circular shape, and with a streak of horizontal pixels resembling a “comet tail”. Note that in this image the transparency slider was positioned halfway so that both the emCCD image and photographic reference image can be seen. (c) Intact WLE image showing a white square artefact. The image shows a very high, uniform brightness which prevents the identification of any details within the image. (d) Lymph node image showing a ring artefact. The dark can be seen. (c) Intact WLE image showing a white square artefact. The image shows a very high, uniform brightness which prevents the identification of any details within the image. (d) Lymph node image showing a ring artefact. The dark rings cover any relevant structure within that area, thus making the image unevaluable. [file 13550_2021_759_MOESM1_ESM.png]
