# Supplementary material for: Intraoperative [18F]FDG flexible autoradiography for tumour margin assessment in breast-conserving surgery: a first-in-human multicentre feasibility study
Source: EJNMMI Res. 2021 Mar 18;11:28. doi: 10.1186/s13550-021-00759-w (PMC7973336; doi:10.1186/s13550-021-00759-w)
Supplement: Supplementary file 2 — Additional file 2: Table 1. Confusion matrix of FAR imaging for intraoperative margin assessment of intact WLE specimens compared to gold standard histopathology. The patients were divided into two subgroups based on the time between [18F]FDG injection and intraoperative FAR imaging (threshold: 158 min). The mean injection to imaging times between the two subgroups was not statistically significant (p < 0.353). [file 13550_2021_759_MOESM2_ESM.docx]

**Supplemental table 1**. Confusion matrix of FAR imaging for intraoperative margin assessment of intact WLE specimens compared to gold standard histopathology. The patients were divided into two subgroups based on the time between [^18^F]FDG injection and intraoperative FAR imaging (threshold: 158 minutes). The mean injection to imaging times between the two subgroups was not statistically significant (p<0.353).

|  |  | **Histopathology +** | **Histopathology -** |
| --- | --- | --- | --- |
| *Late* | **FAR +** | 4 | 28 |
|  | **FAR -** | 2 | 89 |
| *Early* | **FAR +** | 2 | 40 |
|  | **FAR -** | 5 | 215 |
