# Supplementary material for: Intraoperative [18F]FDG flexible autoradiography for tumour margin assessment in breast-conserving surgery: a first-in-human multicentre feasibility study
Source: EJNMMI Res. 2021 Mar 18;11:28. doi: 10.1186/s13550-021-00759-w (PMC7973336; doi:10.1186/s13550-021-00759-w)
Supplement: Supplementary file 3 — Additional file 3: Table 2. Confusion matrix of FAR imaging for intraoperative margin assessment of intact WLE specimens compared to gold standard histopathology. The patients were divided into three subgroups based on T-classification. [file 13550_2021_759_MOESM3_ESM.docx]

**Supplemental table 2**. Confusion matrix of FAR imaging for intraoperative margin assessment of intact WLE specimens compared to gold standard histopathology. The patients were divided into three subgroups based on T-classification.

|  |  | **Histopathology +** | **Histopathology -** |
| --- | --- | --- | --- |
| *T1* | **FAR +** | 5 | 51 |
|  | **FAR -** | 5 | 224 |
| *T2* | **FAR +** | 1 | 15 |
|  | **FAR -** | 2 | 70 |
| *T3* | **FAR +** | 0 | 0 |
|  | **FAR -** | 0 | 0 |
